# 

**Published:** 1881-03

**Authors:** 


CONTENTS:

| ORIGINAL. | | Page. |
|---|---|---|
| The Law of The Similars, | Editorial, | 81 |
| Fatal Errors, | Ad. Lippe, M.D., Phila. | 87 |
| Is Hahnemann Obsolete? | B. H. Cheney, M.D., New Haven, Con. | 90 |
| Similia vs. Idem, | C. Lippe, M.D., New York. | 94 |
| The Study of the Materia Medica, | E. J. L. | 96 |
| A Gem, | | 100 |
| Viburnum Opulus, a proving, | Prof. H. C. Allen, M.D., Ann Arbor. | 101 |
| A Differential Diagnosis of Merc. Iod. Ruber and Merc. Iod. Flav., in Diphtheria, | C. Carleton Smith, M. D., Phila. | 105 |
| The Hering Memorial, | | 108 |
| CLINICAL CASES: | | |
| Clinical Reflections, | Ad. Lippe, M. D, Phila. | 110 |
| Quinsy, | Walter M. James, M.D-, Phila., | 114 |
| Kali Carb,—Rheumatism, | E. W. Berridge, M. D., London. | 117 |
| Syphilinum, in Kidney Disease, &c. | Frances Burritt, M.D., New York. | 120 |
| A Sad Case. | | 122 |

